# Unveiling the Mode of Action of Two Antibacterial Tanshinone Derivatives

**DOI:** 10.3390/ijms160817668

**Published:** 2015-07-31

**Authors:** Dongdong Wang, Wuxia Zhang, Tingting Wang, Na Li, Haibo Mu, Jiwen Zhang, Jinyou Duan

**Affiliations:** College of Science, Northwest A&F University, Yangling 712100, Shaanxi, China; E-Mails: wangdd@nwsuaf.edu.cn (D.W.); wuxia@nwsuaf.edu.cn (W.Z.); 2013051706@nwsuaf.edu.cn (T.W.); 2009010186@nwsuaf.edu.cn (N.L.); sdnw2528@sina.com (H.M.); nwzjw@163.com (J.Z.)

**Keywords:** tanshinone, pyrrolidine, antibacterial, reactive oxygen species (ROS), membrane

## Abstract

In this study, 2-(*N*-pyrrolidine-alkyl) tanshinones bearing pyrrolidine groups were synthesized and the antibacterial mechanism was explored. These derivatives selectively elicited antibacterial activity against Gram-positive bacteria. Moreover, their antibacterial activities were time-, concentration-dependent and persistent. It appeared that Fenton-mediated hydroxyl radicals were involved, and the disruption of cell membranes was observed. This study indicates that 2-(*N*-pyrrolidine-alkyl) tanshinones might be potential candidates as antibacterial agents.

## 1. Introduction

Antibiotic resistance has been a great concern due to the extensive clinical use of classical antibiotics. Thus, the development of new classes of antibiotics has become extremely important. Among all the possible candidates, natural products have attracted increasing research and clinical interest [1,2].

**Figure 1:**
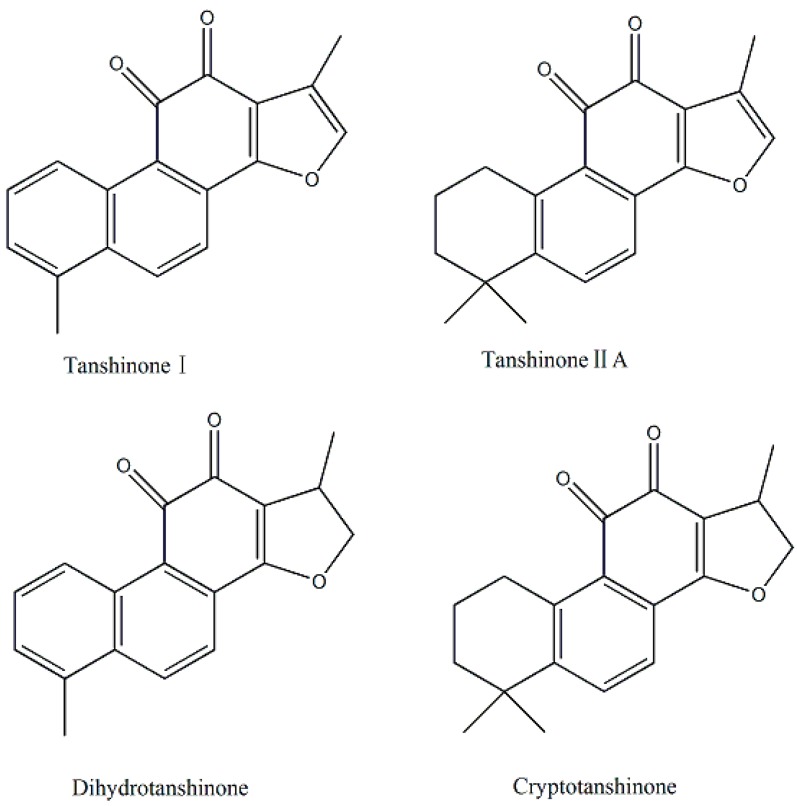
Chemical structures of tanshinone I, tanshinone IIA, dihydrotanshinone and cryptotanshinone.

Tanshinones are a class of abietane diterpene compounds isolated from *Salvia miltiorrhiza* (Danshen), a well-known herb in Traditional Chinese Medicine (TCM) [3,4]. Since they were first identified in the 1930s [5], more than 40 lipophilic and structurally related compounds have been isolated from Danshen. Among the compounds, tanshinone IIA (Tan IIA), tanshinone I (Tan I), dihydrotanshinone and cryptotanshinone (Figure 1) are the four most extensively studied [6]. Cryptanshinone and dihydrotanshinone exhibit antibacterial activity against a broad range of Gram-positive bacteria, however, Tan IIA and Tan I are not toxic to bacteria [7,8]. Tanshinones are generally composed of four rings, including naphthalene or tetrahydronaphthalene ring **A** and **B**, an *ortho*-quinone ring **C**, and furan or dihydrofuran ring **D** [9,10]. The unique structures with potential therapeutic functions have sparked much interest among researchers, and a series of derivatives with modification in ring **D** have been synthesized [11]. Sodium tanshinone IIA sulfonate is a water soluble derivative of tanshinone IIA and has been used in China as a treatment for angina pectoris and myocardial infarction. Moreover, saturated fatty acid, unsaturated fatty acid, or *N*-containing substituent at α-position of furan ring **D** were synthesized [9,12,13]. Additionally, it was reported that alkylated pyrrolidine-modified quinones increase serum half-life and potency against Gram-positive bacteria [14,15]. Thus, 2-(*N*-pyrrolidine-alkyl) tanshinones bearing pyrrolidine groups were synthesized and the antibacterial mechanism was explored.

According to the Mannich reaction, the active hydrogen atom at α-position of furan ring **D**, an aldehyde component (formaldehyde solution), and an amine reagent (pyrrolidine) led to the Mannich base of 2-(*N*-pyrrolidine-alkyl) tanshinone IIA (compound **A**) and 2-(*N*-pyrrolidine-alkyl) tanshinone I (compound **B**) [16]. Using the tanshinone derivatives, we examined the potential antibacterial activity and investigate the mechanism of the antibacterial activity on *Staphylococcus aureus* [17,18,19].

## 2. Results and Discussion

### 2.1. Preparation of Compound **A** and Compound **B**

**Scheme 1:**
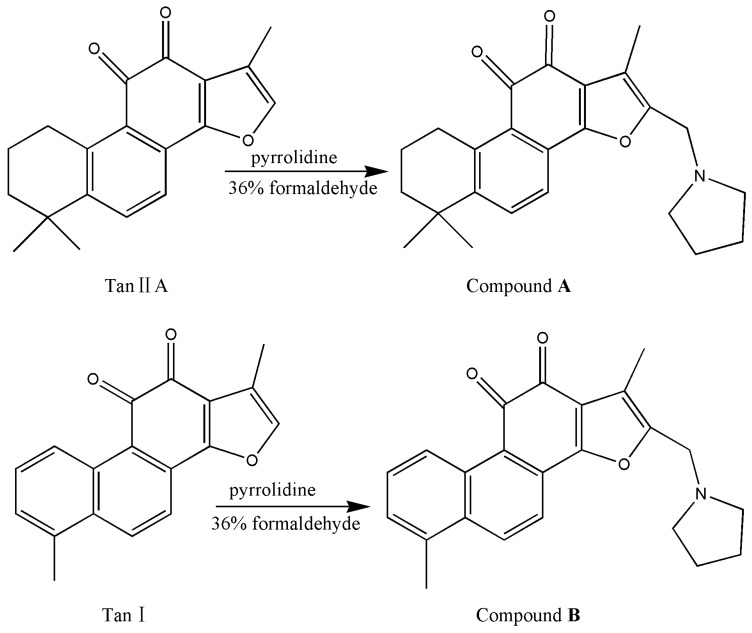
Synthesis of compound **A** and compound **B**.

The synthesis pathway leading to the title compounds is depicted in Scheme 1. Compound **A** or **B** was achieved by Mannich reaction according to the published method [11,20], the structures were elucidated by ^1^H NMR, ^13^C NMR and Mass Spectrometry (MS), which are in agreement with a previous report [11]. For the two compounds, the signal of the active hydrogen atom of the furan ring was at 7.25 ppm (Figures S5 and S6), the disappearance of signals at 7.25 ppm and the appearance of 3.70–3.77 ppm (CH_2_N), 1.74–1.94 ppm (pyrrolidine C_1_, C_4_H) and 2.65–2.74 ppm (pyrrolidine C_2_, C_5_H) (Figures S1 and S3) in the compounds confirmed the synthesis of compound **A** or **B** successful.

### 2.2. Determination of Minimal Inhibitory Concentration (MIC) and Minimal Bactericidal Concentration (MBC)

With compound **A**, compound **B**, Tan IIA and Tan I in hand, the antibacterial activity was evaluated using the broth microdilution susceptibility method against the reference strains of Gram-positive and Gram-negative bacterial species [21].

MIC is reported as the lowest concentration (μg/mL) of drug that visually inhibits growth of the organism [22]. MBC is determined subsequent to MIC testing by sub-culturing diluted aliquots from wells that fail to exhibit macroscopic growth, and is defined as the lowest concentration (μg/mL) of compound exhibiting 99.9% kill over the same time period used to determine MIC. MBC values 16 times higher than MIC typically indicate antibacterial tolerance [23]. The MBC was determined based on Colony Forming Units (CFU) enumerated from Tryptic Soy Broth (TSB) agar plates. If the compound lacks bactericidal activity, the CFUs are too numerous to count and thus reported not determined (n d).

As shown in Table 1, Tan IIA and Tan I did not exhibit antibacterial activity for both Gram-positive and Gram-negative bacteria (MIC > 128 μg/mL). Compound **A** and **B** selectively inhibited Gram-positive bacteria, with the MIC from 8 to 16 μg/mL, however, the compounds were not toxic to Gram-negative bacteria (MIC > 64 μg/mL). The MBC of compound **A** and **B** for the Gram-positive bacteria ranged from 2× to 8× MIC (compound **A** for *Listeria monocytogenes*), which indicated that the bacteria were susceptible to the compounds. Taking account of the value of MBC/MIC, *Staphylococcus aureus* (*S*. *aureus*) was used for the following experiments.

**Table 1:** Antibacterial activity of Tan IIA, Tan I, compound **A** and compound **B** against Gram-positive and Gram-negative bacteria.

MIC/MBC (μg/mL)					
Gram Category	Microorganisms	Tan IIA	Compound A	Tan Ι	Compound B
G^−^	*E. coli*	>128/n d	>64/n d	>128/n d	>64/n d
	*P. aerurginosa*	>128/n d	>64/n d	>128/n d	>64/n d
	*S. typhimurium*	>128/n d	>64/n d	>128/n d	>64/n d
G^+^	*S. aureus*	>128/n d	16/64	>128/n d	8/16
	*L. monocytogenes*	>128/n d	16/128	>128/n d	8/16

G^−^: Gram-negative bacteria; G^+^: Gram-positive bacteria; MIC: minimum inhibitory concentration; MBC: minimum bactericidal concentration; n d: not determined.

### 2.3. Time-Kill Assay

To obtain some insight and information on the mode of action of the antibacterial compounds, compound **A** and **B** were selected to conduct complementary lethality assays. The study is useful for the determination of whether the agents produce concentration-dependent or time-dependent killing and whether the agents have antibacterial or bactericidal profile [24,25].

**Figure 2:**
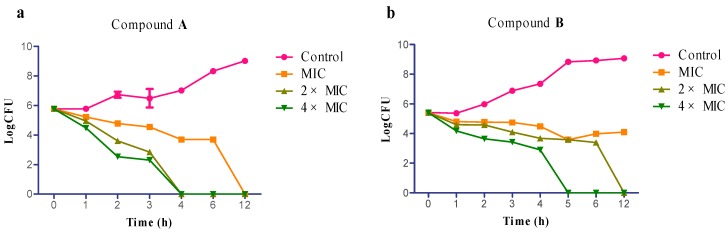
Time-kill assays. Time-kill curves performed in Tryptic Soy Broth (TSB) at 1×, 2×, 4× MIC levels of compound **A** (**a**), compound **B** (**b**) against the *Staphylococcus aureus.* All experiments were performed in triplicate.

The time-kill curves of compound **A** and **B** against *S. aureus* are represented in Figure 2. For both compound **A** and **B**, the killing kinetics were time-dependent and concentration-dependent. Compound **A** was bactericidal at 2× and 4× MIC levels, with a time to bactericidal killing of about 4 h (Figure 2a), and the bactericidal killing also observed in 12 h at the concentration of 1× MIC. Similar results were observed for compound **B** at 2× and 4× MIC levels, with bactericidal killing observed at five hour and 12 h points. Additionally, cells treated with compound **B** at the concentration of 1× MIC did not exhibit killing within 12 h (Figure 2b) [26]. The results indicate that compound **A** and **B** were bactericidal agents and they produced concentration-dependent and time-dependent killing.

### 2.4. Evaluation of Postcontact Effect (PCE)

**Figure 3:**
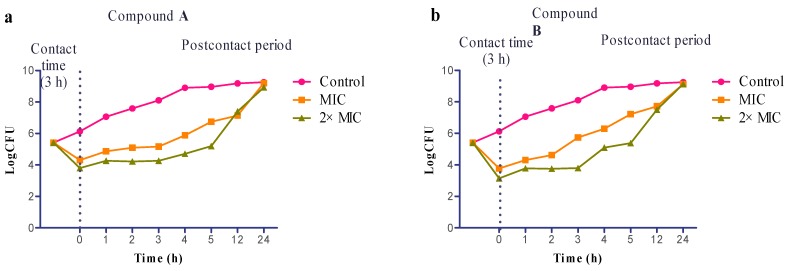
Evaluation of postcontact effect (PCE). The PCE of compound **A** (**a**), compound **B** (**b**) on *S. aureus* growth. All experiments were performed in triplicate.

To assess whether the antibacterial effects of compound **A** and **B** are persistent, we investigated Postcontact Effect (PCE) on *S. aureus*. PCE, derived from the Postantibiotic Effect (PAE), is used to measure the suppression of microbial growth that persists after antibacterial exposure [27,28]. Viable counts decreased after exposure to the two compounds at the concentration of 1× MIC and 2× MIC and did not increase for at least 3 h thereafter. PCE values were both about 2 h at 1× MIC for compound **A** and **B**, about 3 h for compound **A** and 4 h for compound **B** at 2× MIC (Figure 3), showing a sensitive slowing in growth. Bacterial growth increased after 5 h, but the CFU/mL value of pretreated cultures at less than 24 h were lower than the untreated cultures. These data confirmed the bactericidal activity of the agents and the PCE experiments showed that exposure to compound **A** and **B** had a mild persistent antibacterial effect at the time points investigated. This may reflect that the anti-staphylococcal effects with these compounds is mediated by non-specific interactions with membrane bilayers. This also showed a mild effect on the growth rate of surviving bacteria, which had not recovered within 24 h from the end of exposure [29].

### 2.5. Involvement of Hydroxyl Radical in Staphylococcus aureus Inactivation

**Figure 4:**
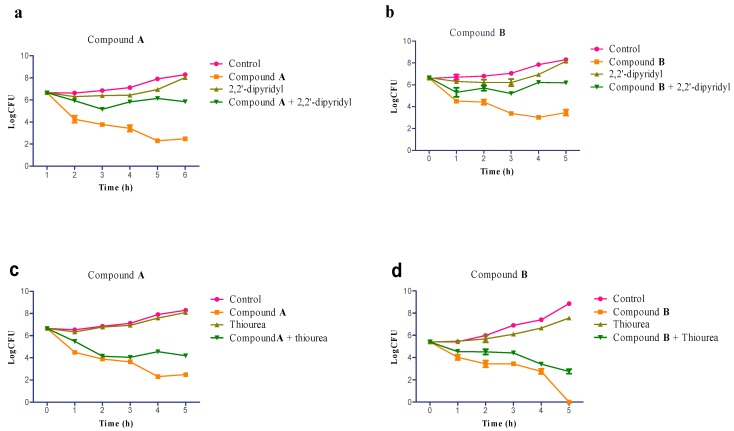
Involvement of hydroxyl radicals in *S. aureus* inactivation. The survival counts of cells treated with compound **A** (32 μg/mL) or compound via **B** (32 μg/mL) following the addition of 500 μM 2,2′-dipyridyl (**a**,**b**) or 150 mM thiourea (**c**,**d**). All experiments were performed in triplicate.

To demonstrate that hydroxyl radical formation is an important component of compound **A**-, **B**-mediated killing, *S. aureus* exposed to compound **A** or **B** were treated with or without the iron chelator, 2,2′-dipyridyl and the radical quencher, thiourea [30,31]. Each experiment was determined in triplicate, and the average of CFU/mL is presented in Figure 4.

For the two bactericidal agent treatments, we observed a significant increase about 3log CFU/mL (Figure 4a,b) in bacterial survival following the addition of 2,2′-dipyridyl, confirming that the Fenton-mediated hydroxyl radicals are involved in compound **A**- and **B**-induced cell death. Reduced hydroxyl radical formation significantly increased the bacterial survival. To directly block the harmful effects of hydroxyl radicals generated via the Fenton reaction, the radical quencher thiourea was added to the treated cultures. The cultures exposed to thiourea with compound **A** or **B** showed the delay in cell death at 1 h and about 3 log CFU/mL increase in survival at 3 h relative to the bactericidal agent treatments alone (Figure 4c,d). The results with 2,2′-dipyridyl and thiourea indicated that hydroxyl radical formation and the Fenton reaction played a critical role in effective killing by compound **A** and **B**. However, thiourea was less efficient at mitigating bacterial cell death after the compounds treatment, which was reflected by the capacity of thiourea to reduce, but not eliminate, the compound **A**-, **B**-mediate hydroxyl radical formation, and this requires further investigation [18,32].

### 2.6. Scanning Electron Microscope (SEM)

**Figure 5:**
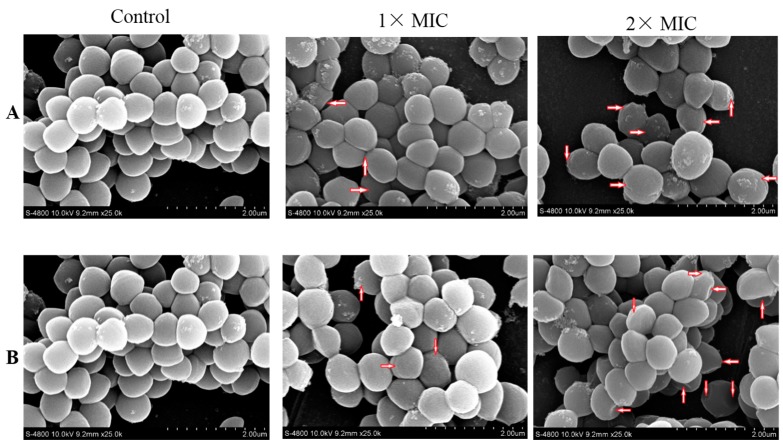
The Scanning Electron Microscope (SEM) photography of *S. aureus*. Untreated bacteria cultured for 3 h (**A**,**B**); bacteria treated with compound **A** at 1×, 2× MIC (A1, A2); bacteria treated with compound **B** at 1×, 2× MIC (B1, B2). The experiment were performed two times with similar results each time. The red arrows represent the observed morphological changes.

*S. aureus* was treated with the compounds at 1× and 2× MIC for 3 h, and the morphological changes were observed by Scanning Electron Microscope (SEM). Figure 5 showed that the untreated bacterial cells exhibited bright and smooth surface with no visible damage, whereas morphological alternations were spotted on the surface of the treated cells, especially at the concentration of 2× MIC of compound **A** and **B**. The treated cells became irregular, pitted, and shriveled (marked by the red arrows). The morphological alternations on the surface might result in the leakage of contents of the cells.

### 2.7. Cell Constituents’ Release

The bacterial membrane serves as a structural component which may be compromised during biocidal challenges, such as exposure to some antibacterial agents. Leakage of cytoplasmic contents is a classic indication of damage to the bacterial cytoplasmic membrane. Therefore, release of intracellular components is a good indicator of membrane integrity. Small ions, such as potassium and phosphate, tend to leach out first, followed by large molecules, such as DNA, RNA and other materials. These nucleotides have strong UV absorptions at 260 nm and are thus described as 260 nm absorbing materials [33,34].

**Figure 6:**
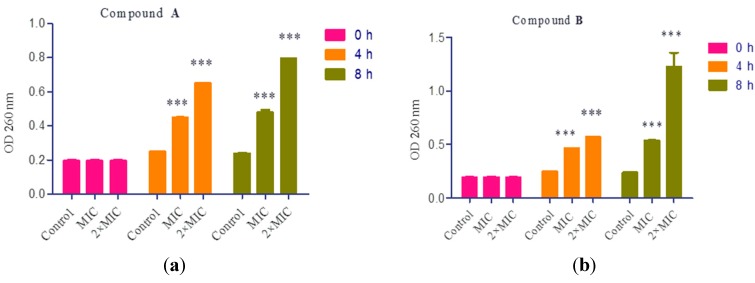
Loss of 260 nm absorbing material. *S. aureus* cells were treated with compound **A** (**a**) and compound **B** (**b**) at 1×, 2× MIC, respectively. All experiments were performed in triplicate. The asterisks denote statistical significance (*p* < 0.05) between the control and the treatments (1× MIC and 2× MIC) of compound **A** and **B** obtained from Tukey’s test. OD: optical density.

The UV absorptions of *S. aureus* culture supernatants are shown in Figure 6, the release of 260 nm absorbing materials from the *S. aureus* quickly increased when treated with compound **A** and **B** at 1× MIC and 2× MIC for 4 h, significantly higher than that for control values. Continuing incubation for another 4 h, the absorbance at 260 nm increased to the higher level. The results indicate that the cell membrane integrity was damaged when the cells were exposed to 1× MIC and 2× MIC of compound **A** and **B**. The alterations correlate with the ability of compound **A** and **B** to interact with hydrophobic structures, such as bacterial membranes [35]. The lipophilic antibacterial agents seem related to direct interaction with the hydrophobic regions of membrane proteins or protein complexes [36,37], resulting in alternation of membrane permeability and leakage of intracellular materials [38].

## 3. Materials and Methods

### 3.1. Chemistry

All reagents were purchased from Aladdin Industrial Corporation and used without further purification, unless otherwise stated. Some solvents were dried prior to use according to standard methods. The reactions were monitored by Thin Layer Chromatography (TLC) on silica gel GF254. Detection was made by both UV light and charring with sulfuric acid/ethanol solution. Column chromatography was performed on silica gel 200–300 mesh. The ^1^H NMR, ^13^C NMR spectra were recorded on a Bruker AV500 MHz (Bruker, Switzerland) instrument using CDCl_3_ as solvents. 

#### 3.1.1. Preparation of Compound **A**

A mixture of tanshinone IIA (29.4 mg, 0.1 mmol), pyrrolidine (15.6 mg, 0.2 mmol), 36% formaldehyde solution (18 mg) in acetic acid (20 mL) was stirred at 20 °C for 1 h, then stirred under reflux at 90 °C for 9 h. Next, the solution was cooled and washed three times with sodium carbonate solution, and extracted with dichloromethane. The organic phase was evaporated and the residue was purified by column chromatography on silica gel (Eluent:ethyl acetate/petroleum ether, 7:10) to produce compound **A** (23.9 mg, 63.5% yield) as orange solid. ^1^H NMR (500 MHz, CDCl_3_) δ 7.65 (s, 2H), 7.31 (s, 1H), 3.71 (s, 2H), 3.22 (t, *J* = 6.4 Hz, 2H), 2.65 (s, 4H), 2.30 (s, 3H), 1.94–1.74 (m, 4H), 1.73–1.65 (m, 4H), 1.35 (s, 6H) (Figure S1); ^13^C NMR (126 MHz, CDCl_3_) δ 183.79, 175.89, 160.63, 150.00, 144.41, 133.36, 127.43, 126.51, 120.59, 120.41, 99.99, 53.98, 49.25, 37.90, 34.67, 31.87, 29.92, 23.51, 19.16, 9.01 (Figure S2); MS (*m*/*z*): calcd for C_24_H_27_NO_3_: 377; found: 378.04 [M + H]^+^ (Figure S7).

#### 3.1.2. Preparation of Compound **B**

A mixture of tanshinone I (27.6 mg, 0.1 mmol), pyrrolidine (15.6 mg, 0.2 mmol), 36% formaldehyde solution (18 mg) in acetic acid (20 mL) was stirred at 20 °C for 1 h, stirred under reflux at 90 °C for 9 h. Next, the solution was cooled and washed three times with sodium carbonate solution, extracted with dichloromethane. The organic phase was evaporated and the residue was purified by column chromatography on silica gel (Eluent:ethyl acetate/petroleum ether, 3:5) to produce compound **B** (19.4 mg, 53.9% yield) as purple solid. ^1^H NMR (500 MHz, CDCl_3_) δ 9.30 (d, *J* = 8.8 Hz, 1H), 8.34 (d, *J* = 8.7 Hz, 1H), 7.93 (d, *J* = 8.7 Hz, 1H), 7.60 (d, *J* = 8.8, 7.0 Hz, 1H), 7.40 (d, *J* = 6.9 Hz, 1H), 3.84–3.72 (m, 2H), 2.75 (d, *J* = 9.1 Hz, 4H), 2.42–2.23 (m, 3H), 1.86 (d, *J* = 59.3 Hz, 4H), 1.36–1.15 (m, 3H) (Figure S3); ^13^C NMR (126 MHz, CDCl_3_) δ 183.53, 175.75, 160.25, 135.24, 133.70, 132.85, 130.67, 129.57, 128.37, 124.80, 123.23, 120.97, 119.11, 99.99, 53.84, 49.01, 23.49, 19.88, 9.02 (Figure S4); MS (*m*/*z*): calcd for C_23_H_21_NO_3_:359; found: 359.98 [M + H]^+^ (Figure S8).

### 3.2. Biology

#### 3.2.1. Bacterial Strains and Growth Conditions

Gram-positive strains: *Staphyloccocus aureus* (ATCC 29213, Guangzhou, China), *Listeria monocytogenes* (CMCC 54004, Guangzhou, China), Gram-negative strains: *Pseudomonas aeruginosa* (PAO1), *Escherichia coli* (ATCC 35150) and *Salmonella typhimurium* (SL1344) were purchased from the National Center for Medical Culture Collection (CMCC). The strains were cultured in Tryptic Soy Broth (TSB) at 37 °C. Active cultures were prepared by transferring a loop of cells from the TSB agar slant to a flask (50 mL) containing 30 mL TSB. They were then incubated at 37 °C to the logarithmic phase of growth. Culture purity was examined by streaking each culture on plates of TSB Agar. The turbidity of the cell suspensions was measured at 600 nm and adjust to the required concentration using the McFarland standard [39]. All bacterial strains were maintained as glycerol stocks at −80 °C.

#### 3.2.2. Minimal Inhibitory Concentration (MIC) and Minimal Bactericidal Concentration (MBC)

Two-fold dilutions of compound **A** and **B** were prepared in a sterile flat-bottomed 96-well polystyrene microplates in TSB, then bacteria with the final concentration of 1.2 × 10^6^ CFU/mL in TSB per well inoculated at 37 °C for 24 h, and bacteria incubated in TSB containing 0.64% (*v*/*v*) DMSO were used as control. MBC is determined subsequent to MIC testing by sub-culturing diluted aliquots from wells that failed to exhibit macroscopic growth. The sample aliquots were inoculated onto TSB agar plates and subsequently incubated at 37 °C for 24 h. The bacterial colonies were enumerated while the growth was readily apparent.

#### 3.2.3. Time-Kill Assay

Cultures of *S. aureus* (about 6 × 10^5^ CFU/mL) at 37 °C were grown in TSB supplemented with 1×, 2×, and 4× the MIC of compound **A** (MIC = 16 μg/mL) and **B** (MIC = 8 μg/mL), with 0.32% and 0.64% (*v*/*v*) DMSO as controls. The number of CFU/mL was measured from aliquots (0, 1, 2, 3, 4, 6, 12 h) that were serially diluted in sterile saline, planted on TSB agar, incubated at 37 °C, and the colonies were enumerated after 18 h. The time-dependent killing curves were constructed by plotting mean colony counts over time.

#### 3.2.4. Evaluation of Postcontact Effect (PCE)

The cultures of *S. aureus* (about 6 × 10^5^ CFU/mL) were grown in media supplemented with 1×, 2× MIC of the compound **A** and **B** for 3 h at 37 °C, 0.32% and 0.64% (*v*/*v*) DMSO as controls. The cultures were washed three times in sterile saline by centrifugation at 3500 rpm for 20 min and then transferred to fresh TSB before incubation at 37 °C. The number of CFU/mL was measured from aliquots that were serially diluted in sterile saline, and planted on the TSB agar immediately and 1, 2, 3, 4, 5, 12, 24 h after incubation. PCE was determined using the equation:

PCE = t − c
(1)

where t was the time needed for a log increase in counts in treated cultures and c was the time needed for a log increase in counts in untreated cultures. All the experiments were performed in triplicate.

#### 3.2.5. Iron Chelator and Hydroxyl Radical Quenching Experiments

2,2-dipyridyl, dissolved in ethanol (0.5 mol/L), was added to the culture with or without compound **A** and **B** at a final concentration of 500 μM, thiourea in solid form was weighed and added to the culture with or without compound **A** and **B** to achieve a final concentration of 150 mM, the cultures with 0.32% and 0.64% DMSO as controls. All the culture of *S. aureus* was adjusted to approximately 10^7^ CFU/mL and cultured at 37 °C. The number of CFU/mL was measured from aliquots that were serially diluted in sterile saline, planted on the TSB agar immediately and 1, 2, 3, 4, 5 h after incubation. The curves were constructed by plotting mean colony counts over time. 

#### 3.2.6. Scanning Electron Microscope (SEM)

Compound **A** and **B** were added at either the 1× MIC or 2× MIC in the culture of *S. aureus* of 1.2 × 10^6^ CFU/mL, 0.64% (*v*/*v*) DMSO as control. All suspensions were incubated at 37 °C for 3 h, and then centrifuged at 3500 rpm for 20 min. All the cells were washed twice with 0.1 M phosphate buffer saline (PBS, pH 7.4) and fixed with 2.5% glutaraldehyde (*v*/*v*) in 0.1 M PBS at 4 °C for 24 h. Next, the cells were dehydrated using 30%, 50%, 70%, 90%, and 100% ethanol, and then the ethanol was replaced by tertiary butyl alcohol. The cells were dried at “critical point” in liquid CO_2_, and the samples were gold-covered by cathodic spraying before examination [40,41].

#### 3.2.7. Cell Constituents’ Release

The experiments were conducted followed a published method [33]. Briefly, the bacterial cells incubated in TSB were adjusted to about 10^8^ CFU/mL. The bacterial suspensions was centrifuged at 5000 rpm for 10 min, washed twice by using the PBS (pH 7.4) and resuspended in sterile saline. Compound **A** and **B** were added to reach the final concentration at 1× MIC and 2× MIC. About 3 mL media was removed from the flasks after 0, 4, 8 h of incubation at 37 °C. The samples were filtered using 0.22 μm syringe filters to remove the bacteria, and the optical density (OD) of the supernatant at 260 nm was recorded using UV visible spectrophotometer. All tests were performed in triplicate. Mean OD values of respective treatments and vehicle control were compared independently at each time point.

## 4. Conclusions

Natural products have been the single most productive source of leads for the development of drugs because of the unmatched availability of chemical diversity. In practice, semisynthesis is the method of choice for lead optimization. In the current study we have synthesized 2-(*N*-pyrrolidine-alkyl) tanshinone II A and 2-(*N*-pyrrolidine-alkyl) tanshinone I successfully. Their antibacterial properties were evaluated and the antibacterial mechanism was explored. The results revealed that the two tanshinone derivatives have promising antibacterial activity against the Gram-positive bacteria, moreover, Fenton-mediated hydroxyl radicals were involved and the disruption of the cell membrane was observed in the inactivation of Gram-positive bacteria. Moreover, the hydrophobic molecules tend to be non-specific, and the mammalian cells may also be affected by the formation of hydroxyl radicals. These results indicated that 2-(*N*-pyrrolidine-alkyl) tanshinones could serve as a starting point for further optimization and promote the development of new antibacterial agents [35,42].

## Supplementary Materials

Supplementary materials can be found at http://www.mdpi.com/1422-0067/16/08/17668/s1.
