# Supplementary material for: Unveiling the Mode of Action of Two Antibacterial Tanshinone Derivatives
Source: Int J Mol Sci. 2015 Jul 31;16(8):17668–81. doi: 10.3390/ijms160817668 (PMC4581214; doi:10.3390/ijms160817668)
Supplement: Supplementary file 1 [file ijms-16-17668-s001.pdf]

Supplementary Information

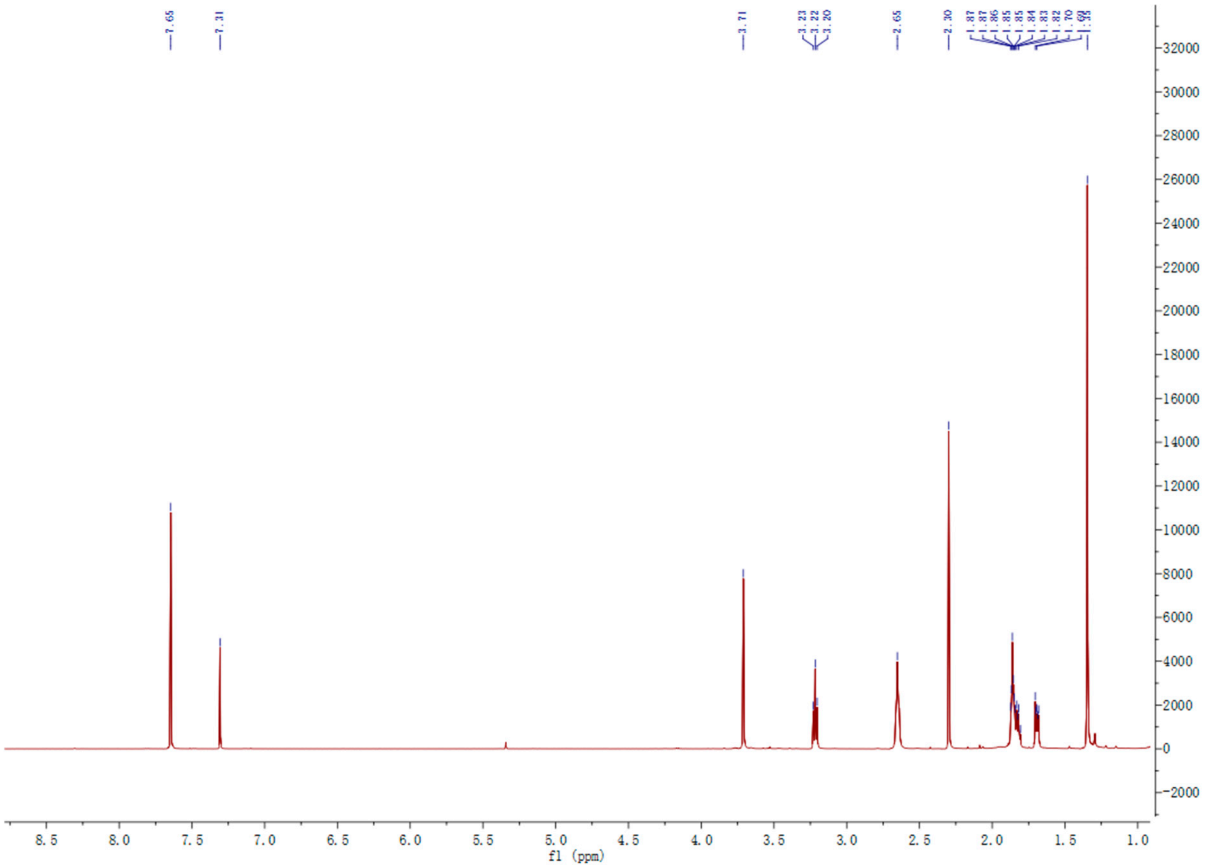

Figure S1. ^{1}H NMR of compound **A**.

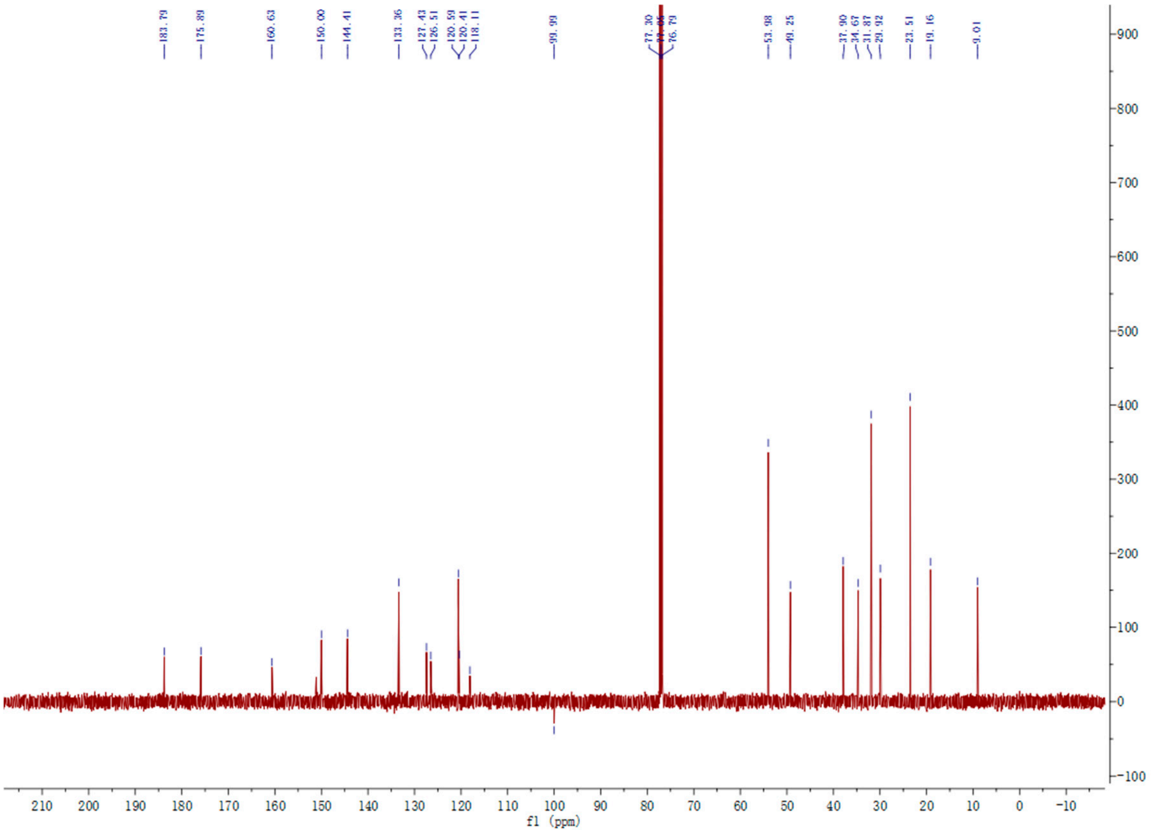

Figure S2. ^{13}C NMR of compound **A**.

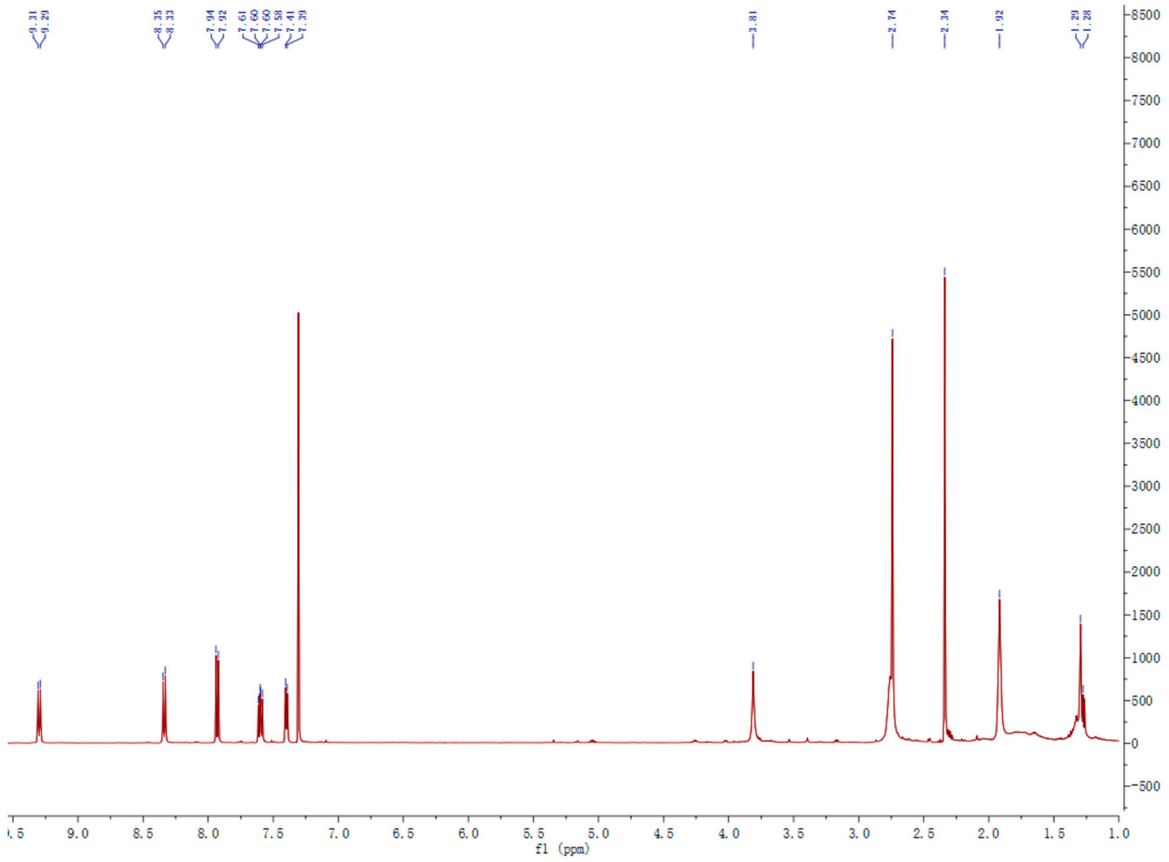

Figure S3. ^{1}H NMR of compound **B**.

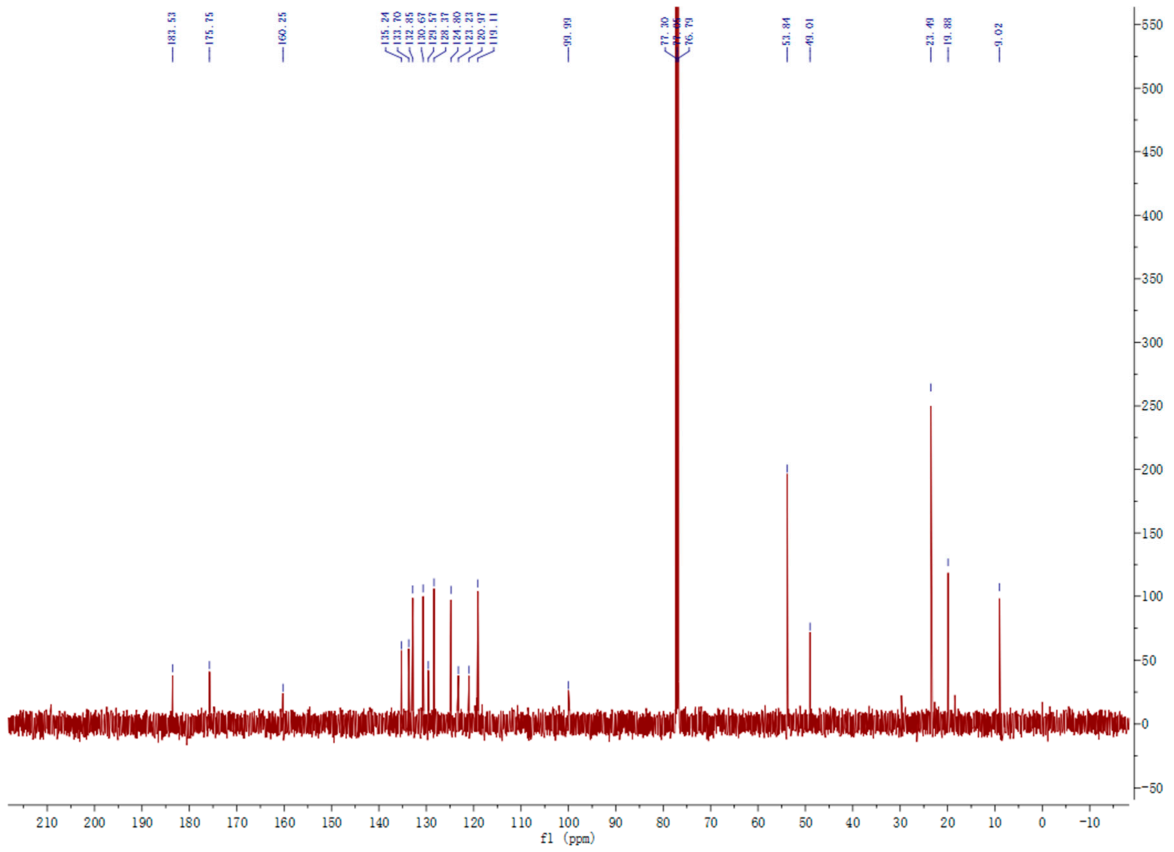

Figure S4. ^{13}C NMR of compound **B**.

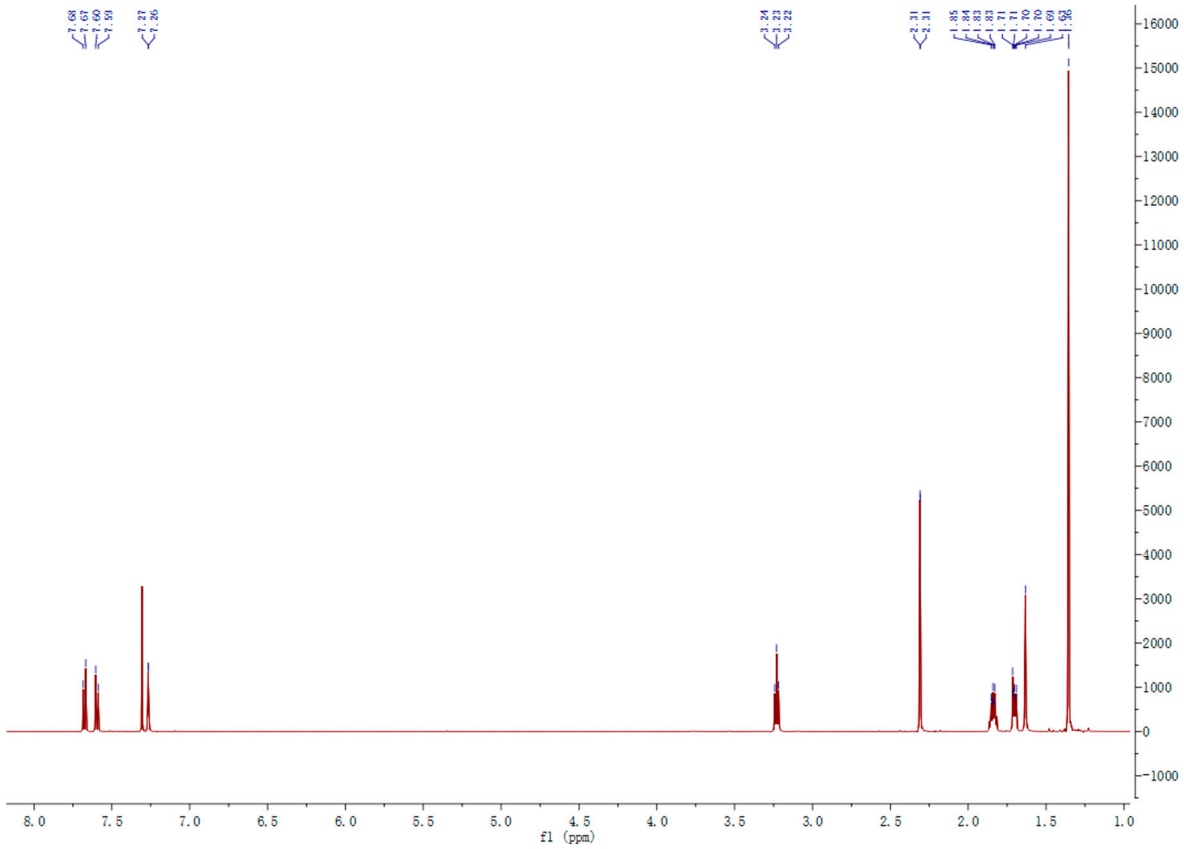

Figure S5. ^{1}H NMR of Tanshinone IIA.

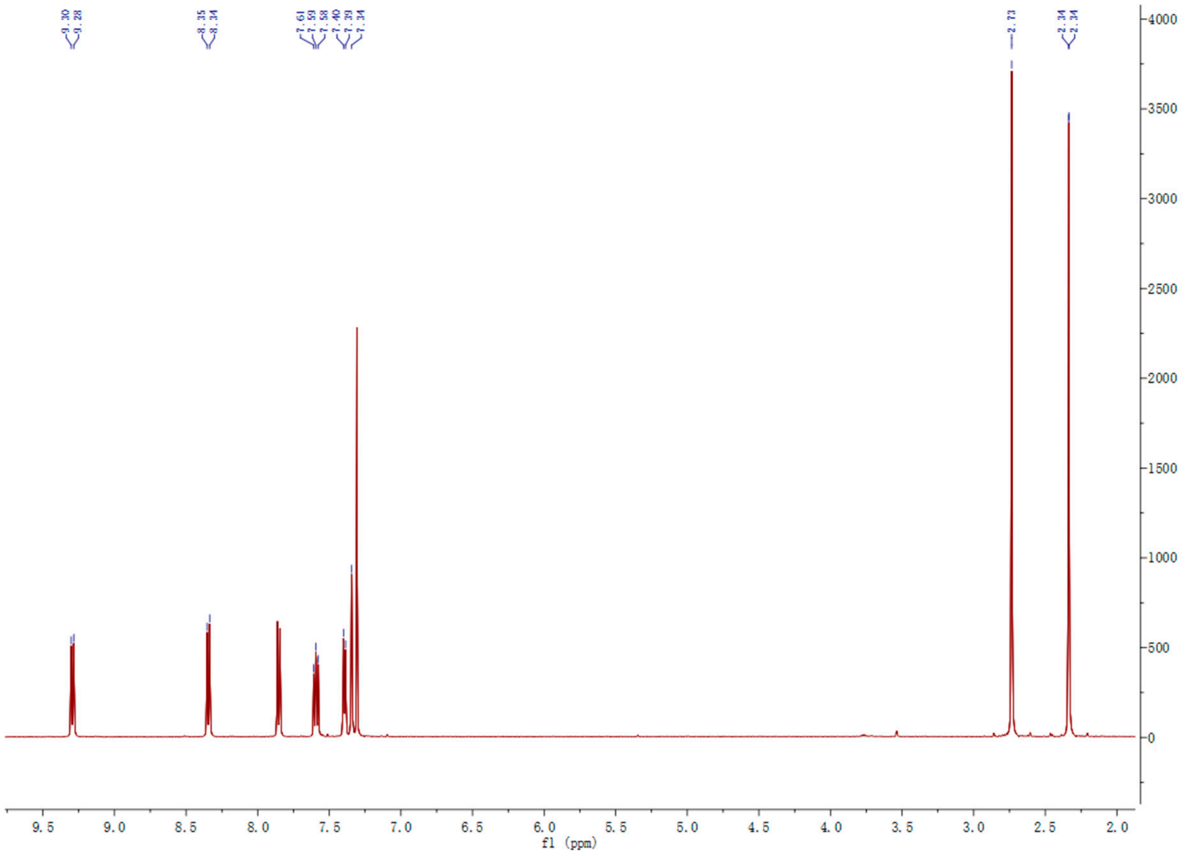

Figure S6. ^{1}H NMR of Tanshinone I.

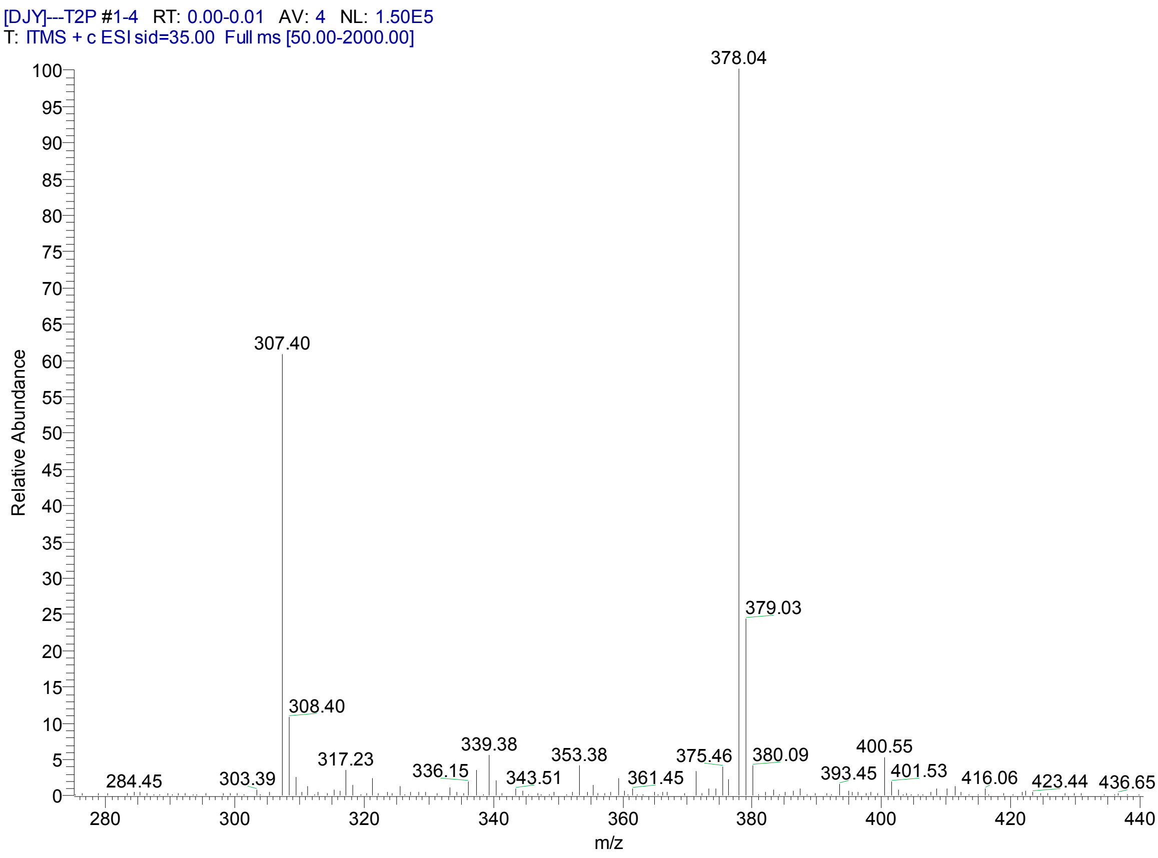

Figure S7. MS of Compound **A**.

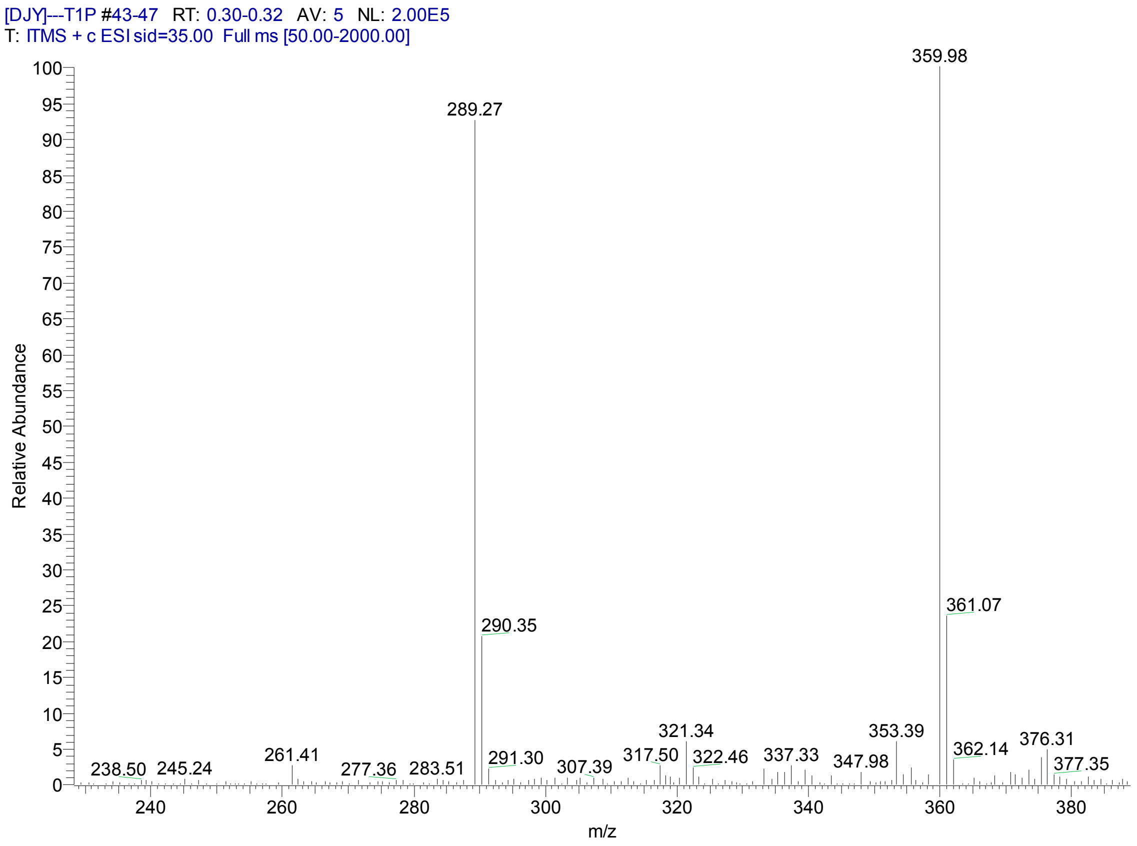

Figure S8. MS of Compound **B**.
